# Ankle joint dislocation treating dislocated trimalleolar fractures accompanied with the complex posterior malleolus fracture without separation of the tibiofibular syndesmosis

**DOI:** 10.1097/MD.0000000000012079

**Published:** 2018-09-14

**Authors:** Wenzhao Xing, Yanfeng Wang, Liang Sun, Linjie Wang, Zhigang Kong, Chunpu Zhang, Zhiguo Zhang

**Affiliations:** a Department of Orthopaedics, Hebei Medical University Third Affiliated Hospital; b Department of Orthopaedics, Affiliated Hospital of Hebei University, Shijiazhuang, China.

**Keywords:** ankle joint dislocation, dislocated trimalleolar fracture, posterior malleolus fracture, the tibiofibular syndesmosis

## Abstract

To explore the therapy of ankle joint dislocation treating dislocated trimalleolar fractures accompanied with the complex posterior malleolus fracture without separation of the tibiofibular syndesmosis and improve surgical results.

Patients who had dislocated trimalleolar fractures accompanied with the complex posterior malleolus fracture without separation of the tibiofibular syndesmosis were retrospectively analyzed and 30 patients were enrolled the study. They were all treated by ankle joint dislocation and the surgical results were evaluated by the Baird–Jackson ankle scoring system. Longitudinal curved incision in medial malleolus was made in ankle joint dislocation and subluxation was automatically formed by appropriate traction of ankle joint. The talus and the distal end of internal and external malleolus were pushed the outside to form the lateral dislocation of the ankle joint. After fully revealed the posterior malleolus and distal articular surface of the tibia, the anatomical reduction of comminuted bones with joint cartilage and posterior malleolus was achieved by fixed with absorbable screw or Kirschner wire. Internal and external malleolus fracture was fixed by the conventional approach.

The average follow-up period was 13 months. According to the Baird–Jackson ankle scoring system, the excellent and good result was 28 cases, fair was 2 cases which the excellent and good rate was 93.3% without talar necrosis in any cases.

Ankle joint dislocation can achieve the satisfactory results in treating dislocated trimalleolar fractures accompanied with the complex posterior malleolus fracture without separation of the tibiofibular syndesmosis. Ankle joint dislocation can make joint cartilage restore accurately with excellent results.

## 1 Introduction

The ankle joint or the talocrural region^[1]^ is the region where the foot and the leg meet.^[2]^ The ankle joint is the weight-bearing joint of the human body, which the stability and flexibility of the ankle joint is very important for the movement of the human body. Ankle fracture is a common clinical fracture, accounting for about 3.9% of the total body fractures. The number of patients with ankle fractures was increased year by year, ranking the first in the intra-articular fracture.^[3]^ Ankle fracture belongs to the intra-articular fracture, which can be divided into single ankle fracture, bimalleolar fracture, and trimalleolar fracture (TF) according to injury mechanism and severity.^[4]^ Severe situation can accompany with the dislocation and the rupture of inferior tibiofibular syndesmosis. TF, also called as cotton fracture, is a serious type of ankle fracture, which was often accompanied by ligament injury of the ankle and dislocation of the ankle joint.^[5]^ TF is the fracture of medial malleolus, lateral malleolus and anterior or posterior edge of the tibia. The surgery of TF was difficult with poor effect, especially for the comminuted posterior malleolus fracture. The reduction and fixation of this type fracture is still a big problem in the medical profession. Patients often had a certain degree of sequelae after treatment, ranging from walking instability, joint deformity, traumatic arthritis, even to joint stiffness.^[6]^ Surgical treatment for TF has become the consensus of the majority of clinicians, but how to clearly expose the posterior malleolus is still inconclusive.

After years of clinical practice, we found ankle joint dislocation (AJD) treating TF with comminuted posterior malleolus fracture can achieve the goal of clear exposure, exact reduction, and good effect.^[7,8]^ However, this method limited the indications of TF with separation of the tibiofibular syndesmosis in the early stages. With the increase in the number of cases, we found that some of the TF cases of ankle fractures without separation of the tibiofibular syndesmosis also can be treated with AJD.

This study tries to use AJD treating dislocated trimalleolar fractures (DTF) accompanied with the complex posterior malleolus fracture without separation of the tibiofibular syndesmosis in 30 cases from June, 2012 to June, 2014 explore the superiority of AJD to expose the posterior malleolus and improve surgical results.

## 2 Material and methods

### 2.1 General data

Patients who had DTF accompanied with the complex posterior malleolus fracture without separation of the tibiofibular syndesmosis were retrospectively analyzed and 30 patients were enrolled the study in the third clinical hospital of Hebei Medical University from June, 2012 to June, 2014. The average age was 30 years old (22–65) with 19 males and 11 females. Causes of injuries include sprain of ankle (12 cases), falling injury (8 cases), and traffic injury (10 cases). Sixteen cases were accompanied with the posterolateral dislocation of the talus (53.3%), 6 cases with lateral dislocation (20%), and 8 cases with posterior dislocation (26.7%). Twelve cases were manipulation failure. All cases were fresh closed fracture without separation of the tibiofibular syndesmosis and the fracture area of the posterior malleolus was at least 25% of the articular surface which the fractures were all comminuted fracture. Inclusion criteria and exclusion criteria were showed in Table 1. According to the Lauge–Hansen classification, 19 cases were supination-external rotation with sequence IV and 11 cases were pronation-abduction with sequence III.^[9]^ According to the Danis–Weber classification (AO/ASIF), 21 cases were type B and 9 cases were type A.^[10]^ Clinical data are shown in Table 2.

**Table 1:**
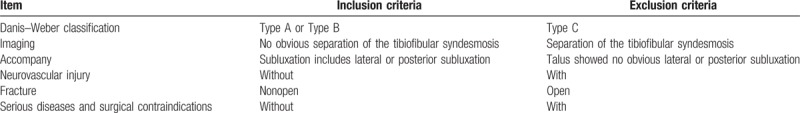
Inclusion criteria and exclusion criteria of the patients.

**Table 2:**
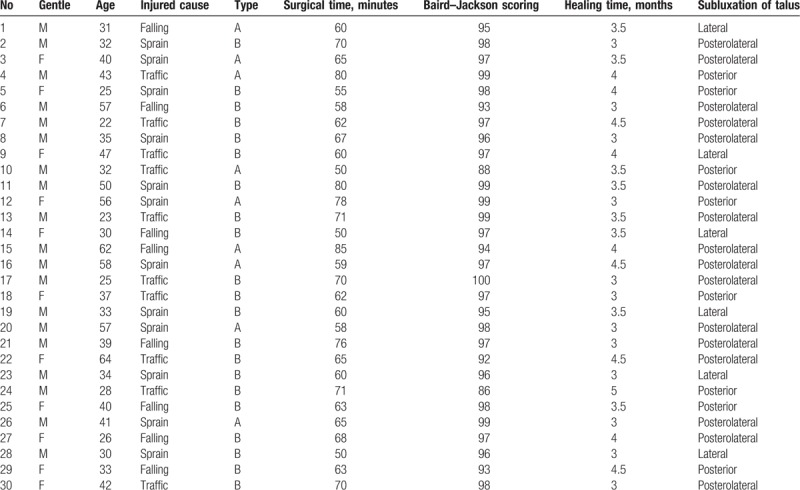
Clinical data of the patients.

This study was approved by the ethics committee at our hospital and was conducted in accordance with the provisions of the Declaration of Helsinki, Good Clinical Practice guidelines, and local laws and regulations. Informed consent had been obtained from participants.

### 2.2 Operation

Patients after admission received swelling treatments such as limb elevation and drug, and routine examinations such as direct and lateral side x-ray photograph and CT examination. After definitely diagnosing the type of fracture, operative proposal was made. Meanwhile, the complications were treated. When the swelling of soft tissue was reduced and without surgical contraindication, the surgery was done. The average time from injury to surgery was 7 days (5–10 days).

**Figure 1:**
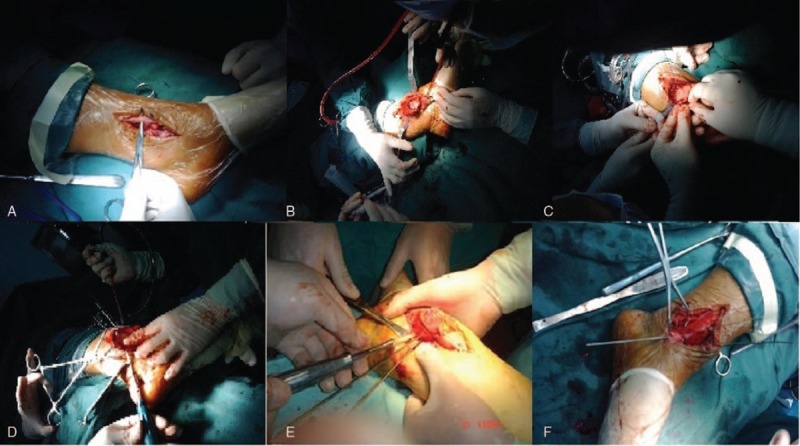
The surgical procedure of the ankle joint dislocation. (A) Inner-side incision. (B) Ankle fracture block and distal articular surface of tibia after dislocation. (A) Free reduction of the posterior malleolus fractures and fixation with Kirschner wire. (D) Main posterior malleolus fracture block fixed by absorbable screw. (A) Removal of temporary fixation (Kirschner wire). (F) Reduction of medial malleolus. ∗The patient was female and 58 years old. Sprain led to swelling and pain of right ankle and she was admitted to hospital 2 day after limiting of activities.

Patient was in supine position, the surgery was done under the electric pneumatic tourniquet, and the surgical field was conventional disinfected. From 2 cm to the medial malleolus tip along with the medical aspect of tibia, 8 cm longitudinal curved incision was made (Fig. 1A). The medial malleolus fracture block was exposed under the protection of great saphenous veins. Periosteum detacher was used to bluntly separate the periosteum to the posterior malleolus, closely to the surface of the tibia, and the medial malleolus fracture block was raised to the far side. By appropriate traction of ankle joint, the talus and the distal end of internal and external malleolus were pushed to the outside forming the lateral dislocation of the ankle joint (Fig. 1B). The posterior malleolus and the distal articular surface of the tibia were fully revealed in the surgical field. Saline gauze was used to protect the articular surface, prolapsed bone and soft tissue. Comminuted bones with joint cartilage were found between the posterior malleolus fractures and the anatomical reduction was achieved by fixed with absorbable screw (Fig. 1C–E). Piecemeal bones were discarded. Ankle joint and medial malleolus fracture block were reducted and fixed with hollow screws or absorbable screws (Fig. 1F). The lateral malleolus adopted fibula lateral incision to expose the fracture broken end and fixed with steel plate after reduction. The wound was washed and sutured.

Prophylactic use of antibiotics was not more than 3 days after the surgery and the suture was removed in 12 to 14 days according to the wound healing situation. Short leg plaster or brace was used to fix for 4 to 6 weeks. Following the principle of “active, progressive and enhanced,” the active exercise of the ankle joint was done after the removal of gypsum. Patients gradually tried to load under the protection 8 weeks later and walk from the 12th week.

### 2.3 Standardization of efficacy evaluation

Efficacy evaluation was evaluated by the Baird–Jackson ankle scoring system,^[11]^ including pain, stability of ankle joint, walking, running, motion range of ankle joint, and ankle x-ray photograph. Excellence was 96 to 100 scores, good was 91 to 95 scores, fair was 81 to 90 scores, and poor was <80 scores.

## 3 Results

Patients in this study were all followed-up and the average time was 13 months (8–24 months). All of patients were treated clinically, healing time for 3 to 5 months (mean: 3.5 months). No talar necrosis happened. According to the Baird–Jackson ankle scoring system, 22 cases were excellent, 6 cases were good, and 2 cases were fair which the excellent and good rate was 93.3%.

The surgical time of this study was 50 to 85 minutes, with an average of 65 minutes. Tourniquet was used during the surgery and the bleeding amount was 60–100 mL. Besides one case with incision healing in phase II, the incision of other cases were all healed in phase I.

**Figure 2:**
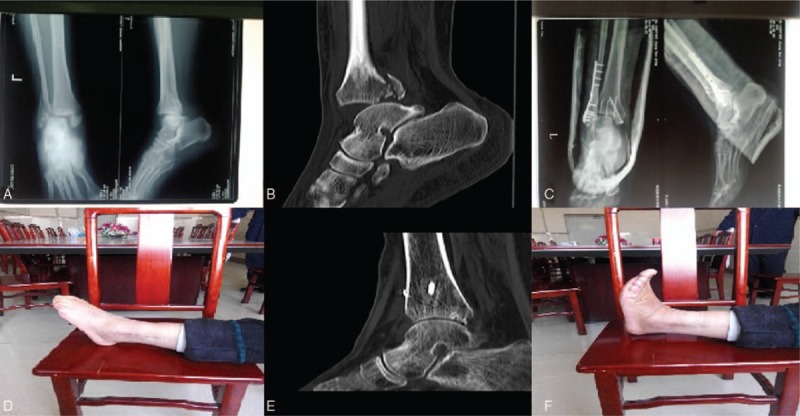
X-ray photograph and CT examination of one patient before and after the ankle joint dislocation. (A) X-ray photograph before the ankle joint dislocation; (B) CT examination before the ankle joint dislocation; (C) X-ray photograph after the ankle joint dislocation; (D) ankle flexion activity one year after the ankle joint dislocation; (E) CT examination 7 months after the ankle joint dislocation; (F) ankle extensor activity one year after the ankle joint dislocation. ∗The patient was female and 58 years old. Sprain led to swelling and pain of right ankle and she was admitted to hospital 2 day after limiting of activities. CT = computed tomography.

No loosening, prolapsed, and broken situation occurred in internal fixation of all cases. All cases had no obvious symptoms of traumatic arthritis of ankle joint (Fig. 2). Six cases expressed that the ankle joint appeared pain symptoms after a large number of activities, which can relief after rest. X-ray photograph showed no obvious signs of traumatic arthritis of ankle joint. Thus, this may be caused by soft tissue adhesion around ankle. After physical therapy and functional exercise for 3 months, the symptoms of 2 cases basically disappeared, the symptoms of 3 cases remitted obviously, and the pain of 1 case was reduced. Ankle joint activity of 28 cases can meet the needs of normal activities, while 2 patients were slightly limited when squatting.

## 4 Discussion

TF is often caused by the strong internal or external rotation violence, which the stable structure of the bone and ligament in the ankle joint was seriously damaged. Most of the fractures are displaced, and the structure of the ankle mortise is damaged, which affects the stability of the ankle joint.^[12]^ Unstable displaced ankle fractures should be treated with open reduction and internal fixation.^[13]^ TF, especially when the posterior malleolus fracture has comminuted blocks, is very difficult to reset. If the reduction is poor, it can easily lead to the occurrence of traumatic arthritis of the ankle joint, affecting the prognosis and life quality of the patients. This situation was called the complex posterior malleolus fracture. For patients with the complex posterior malleolus fracture, the unstable talus dislocation, or the posterior malleolus fracture larger than 25% of the distal articular surface of the tibia, surgical treatment is better than conservative treatment which is not satisfactory.^[14]^ The complex posterior malleolus fracture is characterized by the displacement or compression of the fracture block, the comminution or even the insertion into cancellous bone of the distal articular surface of the tibia.^[14]^ Ankle fractures are intra-articular fractures, and articular surface malunion is easy to cause traumatic arthritis. So we should try our best to achieve anatomical reduction as far as possible in order to prevent and reduce the occurrence of traumatic arthritis. Hartford et al^[15]^ found poor reduction of the distal articular surface of the tibia can significantly increase the pressure of the ankle joint. By three-dimensional finite element study, Goreham-Voss et al^[16]^ discovered if dislocation of articular surface was more than 2 mm, whether fracture is stable or not, the abnormal distribution and peak pressure will appear, resulting in the occurrence of traumatic arthritis. Zhu et al^[17]^ thought when the posterior malleolus fracture was >25% of the distal articular surface of the tibia, talus stability decreased significantly and talus was easy backward dislocation. Thus, smooth articular surface is the key to the anatomical reduction of posterior malleolus fracture, which is also an important criterion to judge the long-term curative effect.

In clinic, we found that lower limbs showed external rotation in some TF patients when the ankle joint was lift for routine disinfection. That meant the ankle joint was prone to lateral dislocation, or even dislocation. After the formation of the medial malleolus incision, the distal medial malleolus fracture blocks were raised, and ankle joint automatically become subluxation. By appropriate traction of ankle joint, the talus and the distal end of internal and external malleolus were pushed to the outside forming the complete dislocation of the ankle joint. This time, the distal articular surface of the tibia can be fully exposed, and the ankle fracture block and the articular surface can be directly reset and fixed. The above TF belongs to severe ankle fracture type, characterized by the complete loss of stability and significant dislocation of the ankle joint. In the Lauge–Hansen classification and AO classification, there was no discussion about DTF.

Through years of clinical observation, the indication of DTF was concluded that TF; ankle joint losing stability with dislocation tendency (or dislocation, or subluxation); separation of the tibiofibular syndesmosis or type A fracture with normal location of the tibiofibular syndesmosis; comminuted posterior malleolus fracture. Lateral dislocation of the ankle joint can be formed as long as the appropriate increase in the valgus strength in DTF, without increasing the damage, and the comminuted fracture blocks can be reset under direct vision. AJD was derived from DTF in the practice. Previous study has proved that AJD treating DTF accompanied with the complex posterior malleolus fracture with separation of the tibiofibular syndesmosis can accurately reset the posterior malleolus comminuted articular surface and the therapeutic effects were good.^[18]^ This study testified AJD also can be used for DTF accompanied with the complex posterior malleolus fracture without separation of the tibiofibular syndesmosis, which the results were excellent.

Advantages of AJD are as follows. No increasing damage. The stable bone structure of the ankle joint had been destroyed in DTF, and talofibular ligament, ligamenta deltoideum, posterior tibiofibular ligament, and capsule on bone no longer played its due role. The ankle joint appeared dislocation or subluxation. The fracture itself has caused the complete damage of the ankle joint. Thus, it is not necessary to destroy the stable structure of the ankle joint for AJD, and will not affect the normal anatomy of the ankle joint. Reduction under direct vision. After the dislocation of the ankle joint, the posterior malleolus articular surface is fully exposed in the surgical field. The comminuted posterior malleolus fracture blocks can be reset and fixed under direct vision. No increasing risk of talus necrosis. Lateral dislocation of the ankle joint will not affect the blood supply of the talus. The main blood supplies of the talus include the tarsal canal artery, the posterior nodal branches of the deltoid branch, superior cervical branches of arteriae tibialis anterior, the tarsal sinus artery, and perforating branch and the posterior nodal branches of the peroneal artery. The above blood vessels distribute in the nonarticular surface of the talus forming invisible artery rings. Dislocation of ankle joint in the surgery only exposed the extremitas anterior of the tibia, without damaging the talar artery rings, so it will not cause talus necrosis.^[19]^ No cases in this study appeared talus necrosis.

At present, there is no gold standard for the diagnosis of the ankle joint fracture accompanied with separation of the tibiofibular syndesmosis. It is necessary to combine clinical examination combined with imaging examination was often used in clinic. De Cesar et al thought physical examination was not reliable for the diagnosis of the tibiofibular syndesmosis injury, but its diagnostic specificity was high. Almost all patients diagnosed with the tibiofibular syndesmosis injury will have positive signs in physical examination. Although CT examination, ultrasound, and MRI are able to judge whether the tibiofibular syndesmosis is injured, it is impossible to determine the need of surgical treatment for the instability of the tibiofibular syndesmosis. CT examination can clearly show the relationship between bone and tibiofibula through the cross section scan and 3D reconstruction, understanding the degree of ankle joint injury and accurately calculating data for the surgical plan. Hermans et al found the diagnostic accuracy of MRI for the degree of tibiofibular syndesmosis injury is higher than that of x-ray photograph, but the higher cost of MRI limits its application. All patients in this study were checked by x-ray photograph and CT examination to prove no separation of the tibiofibular syndesmosis. But one requirement of patients was lateral malleolus fracture under the tibiofibular syndesmosis, which all can use AJD. Whether the tibiofibular syndesmosis is separated are not the contraindications for use of AJD, which was proved by above results. However, we also need relative x-ray photograph, CT examination, and MRI combined with clinical examination to determine the situation of tibiofibular syndesmosis injury and guide our clinical work. However, a weakness in thes studies is small cases with short follow-up period. Perhaps for these reasons, there were no recent complications observed and no potential long-term complications recorded. We will follow up for further.

## 5 Conclusion

DTF accompanied with the complex posterior malleolus fracture without separation of the tibiofibular syndesmosis was treated by AJD, which the surgical procedure is simple, the surgical field is clear, do not aggravate the injury of ankle joint, and do not increase the risk of talar necrosis. Posterior malleolus fracture can achieve anatomical reduction under direct vision with a good treatment effect. However, fewer fractures of this type and fewer cases led to needing further clinical research for complications.

## Acknowledgments

All data and experiments were done by my team. Here we thank Prof. Zhang and Sun for energetic support and help in the process of experience. We really very much appreciate your months of guidance and help.

## Author contributions

**Conceptualization:** Wenzhao Xing, Liang Sun, Zhigang Kong, Zhiguo Zhang.

**Data curation:** Wenzhao Xing, Yanfeng Wang, Liang Sun, Linjie Wang, Zhigang Kong.

**Formal analysis:** Wenzhao Xing, Yanfeng Wang, Zhigang Kong, Chunpu Zhang.

**Funding acquisition:** Yanfeng Wang, Linjie Wang, Zhiguo Zhang.

**Investigation:** Zhiguo Zhang.

**Project administration:** Yanfeng Wang, Chunpu Zhang, Zhiguo Zhang.

**Resources:** Liang Sun, Linjie Wang.

**Writing – original draft:** Wenzhao Xing, Yanfeng Wang, Zhiguo Zhang.
